# Adolescence and online vulnerability: The role of fear of missing out (FoMO): A cross-sectional study during the third wave of the COVID-19 pandemic

**DOI:** 10.1371/journal.pone.0332147

**Published:** 2025-09-15

**Authors:** Marco Giancola, Emanuela Mari, Massimiliano Palmiero, Jessica Burrai, Alessandro Quaglieri, Giulia Lausi, Pierluigi Cordellieri, Angelo Fraschetti, Anna Maria Giannini, Laura Piccardi

**Affiliations:** 1 Department of Biotechnological and Applied Clinical Sciences, University of L’Aquila, L’Aquila, Italy; 2 Department of Psychology, “Sapienza” University of Rome, Rome, Italy; 3 Department of Communication Sciences, University of Teramo, Teramo, Italy; 4 Department of Human and Social Science, Universitas Mercatorum, Rome, Italy; 5 IRCCS San Raffaele Cassino, Cassino (FR), Italy; Tianjin University, CHINA

## Abstract

The use of social networking sites (SNSs) has increased significantly in recent years, particularly among adolescents. These platforms have profoundly reshaped the way adolescents interact with family, peers, and strangers. However, SNSs may also expose users to specific vulnerabilities, such as victimisation, with detrimental effects on mental health and psychophysical well-being. This study examined the relationship between age and online vulnerability, with a focus on the mediating role of fear of missing out (FoMO). A cross-sectional study was conducted with 360 adolescents (mean_age_ = 16.95 years; SD_age_ = 1.29 years; age range = 14–19 years; 183 females). Results indicated that FoMO mediated the association between age and online vulnerability, suggesting that younger adolescents may be particularly susceptible to online vulnerability due to their heightened FoMO. These findings underscore the importance of addressing emotional and psychological factors in efforts to reduce online vulnerability. Implications for internet literacy education and preventative strategies are discussed, along with limitations and future research directions.

## Introduction

Social networking sites (SNSs) are digital platforms that enable individuals and communities to share, discuss, and interact with various types of content, including images, audio, video, news, and editorials, through mobile and web-based technologies [1]. SNSs have become a central feature of adolescents’ daily lives, a trend significantly amplified by global events such as the COVID-19 pandemic [2–5]. This widespread and sustained exposure has prompted growing interest among researchers in examining not only the benefits but also the potential risks of SNSs engagement in youth populations. Indeed, SNSs serve as key spaces for identity exploration, communication with peers and family, and broader community and social participation [6,7]. During the COVID-19 pandemic, they also played a critical role in mitigating feelings of loneliness and social isolation by helping adolescents maintain a sense of social connection and belonging [8].

Nevertheless, alongside these positive aspects, a growing body of research highlighted the risks associated with SNS use, particularly in relation to mental health vulnerabilities in young users [9–14]. These risks span behavioural (e.g., risky self-disclosure, strategic self-presentation), cognitive (e.g., social comparison, altered self-concept, sensitivity to social feedback), and neurobiological domains (e.g., increased stress reactivity, altered reward processing) [15]. Such vulnerabilities raised concerns among educators, parents, clinicians, and policymakers, prompting the need for preventive efforts that promote responsible and informed online behaviour among adolescents [16].

One prominent area of concern is online vulnerability, a construct encompassing a spectrum of harmful outcomes, including exposure to illegal or harmful content, victimisation through bullying or harassment, sharing personal information with strangers, meeting groomed strangers, and accessing sites that promote harmful behaviours [17,18]. Previous research identified several psychological predictors of online vulnerability, such as self-esteem [18], self-disclosure habits [19], network size [20], and network diversity [21]. However, age-related differences in vulnerability, although occasionally reported, have often been relegated to secondary analyses.

Existing evidence suggested that age plays a significant role in susceptibility to online risks. Younger adolescents are more prone to engage in risky online behaviours, such as disclosing personal information without adequate caution [22,23]. While some studies indicated that children aged 8–15 are less likely to interact with strangers on SNSs [22], other studies reported that children aged 7–11 often had online acquaintances they never met in person, with 58% describing these encounters as unpleasant [23]. Similarly, in a study involving Facebook users aged 13–77, Buglass et al. [20] found that age is a negative predictor of online vulnerability, suggesting that younger individuals are particularly at risk.

In addition to the age-related effect on online vulnerability, research indicated that age also influences other factors closely linked to online activity, most notably fear of missing out (FoMO). Defined as “a pervasive apprehension that others might be having rewarding experiences from which one is absent” [24, p. 1841], FoMO reflects a state of constant anxiety, in which individuals become worry with the possibility of missing opportunity for social interactions, meaningful experiences, or other gratifying social events [25]. These worries involve staying constantly connected with what others are doing and are closely linked to the use of SNSs [24].

Previous studies consistently showed that younger individuals reported higher levels of FoMO, albeit with small-to-medium effects sizes (with *r* ranging from −.16 to −.17) [26–29], and some exhibited stronger associations (e.g., *r* = −.39) with no significant sex differences [20,27,30]. These age-related differences reflect developmental changes in the sense of self-assurance and emotional stability that can weaken the need for external validation and social comparison underpinning FoMO [31]. Specifically, as individuals grow up, they tend to develop more stable social relationships, which helps reduce feelings of social exclusion or missed social opportunities [32]. Furthermore, cognitive and emotional maturity enables prioritising responsibilities and managing time more effectively [33]. This heightened maturity reduces the need for constant connectivity or updates on social events, ultimately decreasing the likelihood of experiencing negative emotions associated with feeling left out of significant shared experiences [34]. Older individuals also tend to prioritise quality over quantity in their social interactions, potentially reducing the levels of FoMO and the need to attend every social activity or event.

Beyond its association with age, FoMO emerged as a key driver of problematic online behaviours among adolescents. High levels of FoMO were linked to increased risk of cyberbullying victimisation, online aggression, and social media stalking, all of which pose significant threats to well-being and a higher exposure to online vulnerability [35,36]. For instance, in a sample of 506 Facebook users (including 291 adolescents aged 13–17), Buglass and colleagues [37] found that FoMO positively predicted online vulnerabilities: receiving critical or hurtful comments, or experiencing stalking or harassment. Other studies showed that individuals with high levels of FoMO tend to be more vulnerable online due to their attempt to compensate for their feelings of social inadequacy. In addition, individuals who are afraid of being left out online attempt to regain a sense of control by sharing extensive information about themselves on their profiles, which can make them more vulnerable [38].

These findings suggest that FoMO may constitute a central psychological mechanism linking age and online vulnerability, particularly during adolescence, a developmental stage characterised by heightened sensitivity to peer influence, social acceptance, and a desire for social validation through online platforms.

## The present study

This study aimed to examine the mediating role of FoMO in the association between age and online vulnerability among adolescents during the third wave of the COVID-19 pandemic in Italy, a period marked by heightened reliance on SNSs to fulfil their affiliative and social needs during the pandemic.

The research was grounded in three main theoretical assumptions. First, age is a critical factor in adolescents’ engagement in risky online behaviours, such as disclosing personal information without foresight [22,23]. Second, FoMO emerges as a developmental by-product of intensified social needs during adolescence, reflecting heightened sensitivity to peer exclusion and unmet rational expectations [39]. Third, FoMO plays a key role in increasing adolescents’ susceptibility to a variety of online vulnerabilities, such as exposure to harmful content, cyberbullying, and the excessive sharing of personal information [37].

Based on these premises, this study proposed a mediation model in which age predicts online vulnerability through FoMO. Specifically, the hypothesis was that younger adolescents report higher levels of FoMO, which in turn predicts greater online vulnerability, compared to their older peers.

## Method

### Participants

The minimum required sample was evaluated by performing an *a-priori* power analysis by G*Power 3.1.9.7 software [40], using the following parameters: test family: “*F* test analysis”, statistical test: “Linear multiple regression: fixed model, *R2* deviation from zero”, type of analysis: “A priori: Compute required sample size – given *α*, power and effect size”, *α* err prob = .05, power (1-*β* err prob) =.95, mean effect size *f2* = .15 (medium effect), and a maximum number of predictors = 3. The G*Power software revealed that the recommended minimum sample size was N = 119.

A convenience sample of 361 Italian adolescents was recruited for this research. One participant was identified as a univariate outlier (outlier threshold: ± 3 *z*-scores), which was excluded from the dataset. The final sample consisted of 360 adolescents aged 14–19 years (mean age = 16.95 years; SD = 1.29 years; 183 females). No missing data were found considering the final sample. If participants were under 18, they were required to state that their legal guardian had given their consent for their participation in the survey. All participants declared to use social media. The Internal Review Board of the University approved this survey in accordance with the Declaration of Helsinki.

### Measures

In addition to collecting demographic information (age and gender), the following scales were administered (see S1 Appendix for the full list of scales used in this study):

1) Fear of Missing Out Scale [24,34]. This scale consists of 10 items (e.g., “I fear others have more rewarding experiences than I”) anchored on a 5-point Likert scale ranging from 1 (not at all true for me) to 5 (extremely true for me). Following previous research [24], a mean score was computed, with higher scores indicating greater levels of FoMO. This scale has demonstrated good psychometric properties across various cultural contexts, including Italian samples [41]. In this study, the internal consistency reliability was acceptable (Cronbach’s *α *= .75).
2) Online Vulnerability Scale [37]. This scale includes 6 items anchored on a 5-point Likert scale ranging from 1 (very rarely) to 5 (very often). The Online Vulnerability Scale assesses the frequently of adolescents’ exposure to different online vulnerabilities while using Facebook, including critical or hurtful comments, social embarrassment, damaging gossip, and rumours, content of sexual or violent nature, unwanted attention and data misuse. A mean score was computed, with higher scores reflecting greater exposure to online vulnerability. The scale showed good psychometric properties in previous studies [37], and demonstrated good internal consistency reliability in the current sample (Cronbach’s *α *= .80).

### Procedure

The data analysed in the present study were part of a larger research project aimed at identifying the main psychological mechanisms underlying youth online vulnerabilities and developing potential prevention guidelines, with a particular focus on online discrimination against women.

Data were collected during the third wave of the COVID-19 pandemic, between 24 February and 14 April 2021, via an online survey. High school teachers were formally contacted to facilitate participant recruitment. Adolescents aged 18 years or older provided informed consent prior to participation. For minors, parents or legal guardians received an information sheet outlining the objectives of the study, procedures, and measures, and written consent was obtained before minors were permitted to participate. The survey was disseminated via a shareable online link distributed across various social networking platforms (e.g., Facebook, WhatsApp, and Instagram), and all responses were collected through the Qualtrics platform (Qualtrics, Provo, UT). Each participant was assigned a unique Qualtrics ID, and the survey was configured to prevent the collection of identifiable information (e.g., names, birth dates, IP addresses), ensuring full anonymity. All participants completed the entire survey, resulting in a dataset with no missing values. Completion times ranged from 8.48 to 12.68 minutes.

### Design

**Fig 1:**
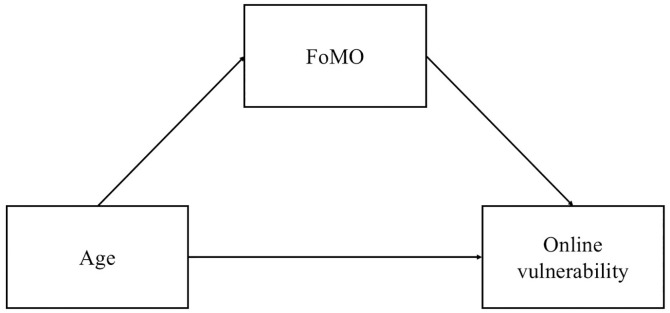
The theoretical mediating model of the current research. FoMO, Fear of missing out.

This research employed a cross-sectional, correlational design to examine the potential mediating role of FoMO in the relationship between age and online vulnerability among adolescents. To test the research hypothesis, we employed a mediation model in which age was the independent variable (x), online vulnerability was the dependent variable (y), and FoMO was the mediator. Fig 1 displays the model proposed in this study.

### Data analysis

Data analyses were conducted using IBM SPSS Statistics for Windows, Version 24.0 (IBM Corp., Armonk, NY, USA). Preliminary analyses employed descriptive statistics and correlational analysis to assess the key characteristics of the variables under study and their interrelationships. The mediating role of FoMO on the association between age and online vulnerability was calculated using the PROCESS macro for SPSS (version 3.5) [42]. The significance of the mediating effects was analysed using 5,000 resamples of bootstrapped estimates with 95% bias-corrected confidence intervals-CIs [43]. The 95% CIs must not cross zero to satisfy the criteria of mediation [43]. Bootstrapping represents one of several resampling strategies for estimation and hypothesis testing. Through bootstrapping, the sample is considered a pseudo-population representing the broader population from which the sample was derived [43]. Additionally, when using bootstrapping, no assumptions about the shape of the sampling distribution are necessary during inferential tests [43]. Bootstrapping is a non-parametric technique that allows for an accurate assessment of the indirect effect in smaller to medium-sized samples while bypassing the issue of non-normality [44–47]. All significance was set to *p* < .05.

## Results

Data were tested for normality, and the analysis showed that the variables of interest were not normally distributed (Kolmogorov-Smirnov Test: Z_Age _= .00, *sig*; Z_FoMO _= .03, *sig*), except for online vulnerability (Kolmogorov-Smirnov Test: Z_Online vulnerability _= .07, *ns*). In order to verify the common method bias (CMB), Harman’s single-factor test [48] was used, showing that the variance explained by a single-factor exploratory model was 30.79. Therefore, the analysis indicated that the data showed no CBM problems (test critical threshold ≥ 50%). As shown in Table 1, the preliminary correlational analysis revealed that age was negatively correlated with FoMO (*r* = –.12, *p* < .05) and positively with online vulnerability (*r* = .12, *p* < .05). This latter was also found to be positively correlated to FoMO (*r* = .13, *p* < .05) and negatively associated with gender (*r* = –.17, *p* < .01).

**Table 1:** Means, standard deviations, and inter-correlations amongst all variables.

	*M*	*SD*	1.	2.	3.	4.
1. Gender			1			
2. Age	16.95	1.29	.07	1		
3. FoMO	2.15	.61	–.03	–.12*	1	
4. Online vulnerability	2.52	.84	–.17**	.12*	.13*	1

*N* = 360, gender was dummy coded (0 = F; 1 = M), FoMO = Fear of missing out.

*p < .05 (two-tailed), ** **p* *< .01 (two-tailed).

**Fig 2:**
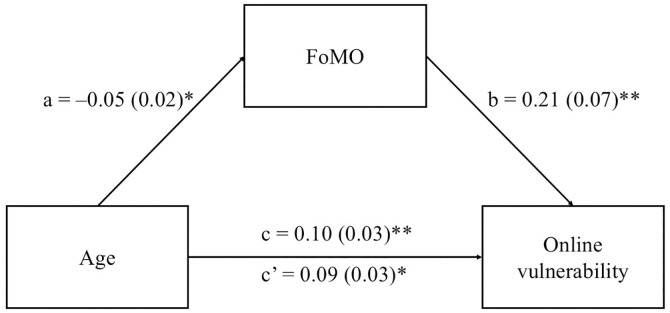
Summary of the results of the mediating model of the current study. FoMO, Fear of missing out. The letters a, b, c, and c’ are path coefficients representing unstandardised regression weights and standard errors in parentheses. ** *p* < .01, * *p* < .05.

After controlling for gender, results revealed that the direct effect of age on online vulnerability was significant (*B* = .10, *SE* = .03, *t* = 3.03, *p* < .01, *CI* 95% = [.036,.168]). Additionally, age was associated to FoMO (*B* = –.05, *SE* = .02, *t* = –2.19, *p* < .05 *CI* 95% = [–.104, –.005]), which in *t*urn predicted online vulnerability (*B* = .21, *SE* = .07, *t* = 3.01, *p* < .01 *CI* 95% = [.074,.352]), suggesting *t*hat FoMO mediated the association between age and online vulnerability (*B* = –.01, *BootSE* = .01, *BootCI* 95% = [–.27, –.001]) (see Fig 2). The total effect (*B* = .09, *SE* = .03, *t* = 2.67, *p* < .05, *CI* 95% = [.024,.156]), and the *R*^*2*^ for the en*t*ire model was.07 [*F*(3,356) = 9.40, *p* < .001]. As the *R*^*2*^ obtained was lower than the medium effect size set in the a priori power analysis (.07 *vs*.15), we performed a new power analysis, finding that the recommended minimum sample size was 250 subjects with a power of.95. This result means that with a sample of 360 subjects, the current study satisfies the recommended minimum sample size. In addition, by performing a post hoc power analysis, the power obtained was.99, suggesting that the power reached by the mediation model was satisfactory.

## Discussion

The present study aimed to investigate the role of FoMO in the relationship between age and online vulnerability among adolescents during the third wave of the COVID-19 pandemic. This period marked a critical phase of the pandemic in Italy, leading the Ministry of Health to enforce stringent health guidelines and restrictions (e.g., social distancing), which significantly disrupted social interactions and relationships [49].

Results indicated a small but consistent mediating effect of FoMO on the relationship between age and online vulnerability, supporting the main research hypothesis. In particular, findings suggested that younger age was associated with higher levels of FoMO (“path a” of the mediation model), which, in turn, positively predicted online vulnerability (“path b” of the mediation model).

As for “path a”, results align with previous research stressing that younger populations, such as adolescents, typically experience elevated levels of fear and anxiety related to the possibility of missing out on social opportunities [26–28]. Indeed, adolescents have a strong desire to remain constantly connected with their peers and stay informed about their activities, which increases the likelihood of socialising regardless of time or place [50]. The impact of the COVID-19 pandemic heightened adolescents’ vulnerability, particularly due to the disruption caused by frequent lockdown measures and the irregular reopening of schools. This forced adolescents to rely heavily on distance learning, potentially amplifying the online challenges they face [49].

Regarding “path b”, the findings showed that FoMO had a positive effect on online vulnerability, indicating that elevated levels of FoMO increase adolescents’ susceptibility to various online vulnerabilities in the social media landscape. This evidence supports previous research that emphasises the role of FoMO in adverse behaviours among adolescents in terms of online aggression or experienced victimisation, such as cyberbullying victimisation, internet aggression, and social media stalking [51]. Similarly, other studies [20] found that adolescents with high levels of FoMO are more exposed to online vulnerabilities on Facebook, including critical or hurtful comments and stalking or harassment.

The analysis of the current study revealed different coefficients for the direct and indirect effects: a positive coefficient for the direct effect and a negative one for the indirect effect. These findings revealed that age plays a twofold role in explaining adolescents’ online vulnerability. On the one hand, the higher age, the higher online vulnerability, suggesting that older adolescents tend to be more vulnerable on SNSs. One explanation for this result could be that younger adolescents mainly interact with peers and schoolmates within specific and controlled social media environments (e.g., learning groups). By contrast, older adolescents have a higher opportunity to explore SNSs (e.g., interacting with strangers or being exposed to harmful social media groups), and consequently, show a high probability of being involved in risky behaviours, such as receiving, downloading, and distributing hateful content as well as being bullied, stalked or harassed [17]. On the other hand, in the presence of FoMO, our findings suggest that younger adolescents, who are more prone to experiencing FoMO, tend to be highly vulnerable when engaging in social media activities. In other words, given that for younger populations, FoMO is a critical psychological factor that deeply influences social relationships, this variable may depict a risk factor for online vulnerability, particularly among younger adolescents.

Overall, this study presents both theoretical and practical implications for the responsible use of the internet. First, this research highlights FoMO as a potential contributor to adolescents’ online vulnerability, such as critical or hurtful comments, social embarrassment, and exposure to sexual or violent content. These findings suggest that concerns about missing out on social opportunities may amplify susceptibility to risks in the social media environment, particularly among younger adolescents. Second, while the mediating effect of FoMO in this research was small, its statistical significance highlights the relevance of addressing the emotional factors underlying online vulnerability. A deeper understanding of these factors may help provide specific guidelines for educating adolescents in internet literacy and advocacy skills. Such guidelines for the younger generation could help reduce the opportunity of being involved in online vulnerabilities and ensure overall positive youth development [52].

Despite these implications, several limitations warrant consideration and offer future research directions. First, the cross-sectional survey design precludes causal inference, as it captures the associations at a single point in time. To address cause-and-effect inferences, future research should employ longitudinal survey designs. Second, the use of a convenience sampling method may limit the generalisability of findings. This method could have underrepresented adolescents facing economic or technological barriers, or those with limited digital access profiles. Moreover, the small but consistent effect size of the indirect effect suggests that the mediating role of FoMO should be interpreted with caution. As a dynamic and developmentally sensitive construct, FoMO may be particularly affected by peer-related pressures, which could moderate its strength across age groups. The modest size of the mediation effect may be due to the socio-emotional context in which the study was conducted (the third wave of COVID-19), which likely heightened anxiety and inflated FoMO levels [53]. Additionally, unmeasured individual differences, such as personality (e.g., Big Five [54–57] and Dark Triad [58,59]), cognitive styles (e.g., field dependence-independence [60,61] and holistic-analytic [62,63]), sociodemographic factors, and online behaviours (e.g., time spent online or on different social media platforms) may have modulated the pathway between age, FoMO, and vulnerability. Finally, as the Online Vulnerability Scale allows only appreciating the potential exposure to vulnerability on Facebook, future research should explore the mediating effect of FoMO, considering a wide array of SNSs, such as Instagram, TikTok, and Reddit.

## Supporting information

S1 Appendix This is the Appendix. (DOCX)
