# Supplementary material for: Adolescence and online vulnerability: The role of fear of missing out (FoMO): A cross-sectional study during the third wave of the COVID-19 pandemic
Source: PLoS One. 2025 Sep 15;20(9):e0332147. doi: 10.1371/journal.pone.0332147 (PMC12435702; doi:10.1371/journal.pone.0332147)
Supplement: S1 Appendix — (DOCX) [file pone.0332147.s001.docx]

**Appendix**

**Fear of Missing Out Scale**

1. I fear others have more rewarding experiences than I

2. I fear my friends have more rewarding experiences than I

3. I get worried when I find out my friends are having fun without me

4. I get anxious when I don't know what my friends are up to

5. It is important that I understand my friends’ “in joke”

6. Sometimes, I wonder if I spend too much time keeping up with what is going on

7. It bothers me when I miss an opportunity to meet up with friends

8. When I have a good time, it is important for me to share the details online (e.g.

updating status)

9. When I miss out on a planned get-together, it bothers me

10. When I go on vacation, I continue to keep tabs on what my friends are doing

**Online Vulnerability Scale**

1. Critical or hurtful comments
2. Social embarrassment involving other users
3. Damaging gossip and rumours
4. Personal data misuse
5. Content of a sexual or violent nature
6. Unwanted attention, stalking, or online harassment
